# Alström Syndrome: Genetics and Clinical Overview

**DOI:** 10.2174/138920211795677912

**Published:** 2011-05

**Authors:** Jan D Marshall, Pietro Maffei, Gayle B Collin, Jürgen K Naggert

**Affiliations:** 1 The Jackson Laboratory, Bar Harbor, Maine, USA; 2 Dipartimento di Scienze Mediche e Chirurgiche, Clinica Medica 3, Azienda Ospedaliera di Padova, Italy

**Keywords:** *ALMS1*, Alström syndrome, ciliopathy, truncal obesity.

## Abstract

Alström syndrome is a rare autosomal recessive genetic disorder characterized by cone-rod dystrophy, hearing loss, childhood truncal obesity, insulin resistance and hyperinsulinemia, type 2 diabetes, hypertriglyceridemia, short stature in adulthood, cardiomyopathy, and progressive pulmonary, hepatic, and renal dysfunction. Symptoms first appear in infancy and progressive development of multi-organ pathology leads to a reduced life expectancy. Variability in age of onset and severity of clinical symptoms, even within families, is likely due to genetic background.

Alström syndrome is caused by mutations in *ALMS1*, a large gene comprised of 23 exons and coding for a protein of 4,169 amino acids. In general, *ALMS1* gene defects include insertions, deletions, and nonsense mutations leading to protein truncations and found primarily in exons 8, 10 and 16. Multiple alternate splice forms exist. ALMS1 protein is found in centrosomes, basal bodies, and cytosol of all tissues affected by the disease. The identification of ALMS1 as a ciliary protein explains the range of observed phenotypes and their similarity to those of other ciliopathies such as Bardet-Biedl syndrome.

Studies involving murine and cellular models of Alström syndrome have provided insight into the pathogenic mechanisms underlying obesity and type 2 diabetes, and other clinical problems. Ultimately, research into the pathogenesis of Alström syndrome should lead to better management and treatments for individuals, and have potentially important ramifications for other rare ciliopathies, as well as more common causes of obesity and diabetes, and other conditions common in the general population.

## INTRODUCTION

Alström syndrome (OMIM 203800) is a rare autosomal recessive genetic disorder, thought to have a prevalence of less than one per million in the general population. It is characterized by the progressive development of multi-organ pathology [1-3]. Symptoms first appear in infancy with great variability in age of onset and severity of clinical symptoms, even within families bearing identical mutations [4]. The severity of the disease, often leading to organ failure, results in a reduced life expectancy, rarely exceeding 50 years. 

Alström syndrome encompasses cone-rod dystrophy in infancy, hearing loss, childhood truncal obesity, hyperinsulinemia and insulin resistance, type 2 diabetes mellitus (T2DM), hypertriglyceridemia, short stature in adulthood, dilated cardiomyopathy (DCM), and progressive pulmonary, hepatic, and renal dysfunction. Fibrosis of unknown etiology develops in multiple organs [4]. The clinical care of individuals is complex due to the combination of multiple endocrine disorders, sensorineural deficits, cardiac, renal, and hepatic abnormalities. There is no specific therapy and individuals are treated and monitored on the basis of individual symptoms. A diagnosis is usually established on the basis of clinical features observed but may be delayed as a result of gradual evolvement and variable expression [2,3]. 

Since Alström syndrome is caused by mutations in the *ALMS1* gene, molecular genetic analysis can be used to confirm the clinical diagnosis [5-7]. The identification of two mutated alleles or a single mutated *ALMS1* allele in the presence of four age-dependent primary features or three primary features plus two age-dependent secondary features are criteria for diagnosis [3]. Heterozygous carriers are asymptomatic, and testing for at-risk relatives, prenatal diagnosis, and preimplantation genetics usually requires prior identification of disease-causing mutations in the family. A screening strategy that first targets the regions of the gene where most of the mutations occur (exons 16, 10, 8) has been successfully used [8-10]. Additionally, the recent development of arrayed primer extension (APEX) technology can be used as an efficient and cost-effective first pass screening tool for known single base substitutions, deletions and insertions in* ALMS1 *and for 10 known genes causing a similarly related disorder, Bardet-Biedl syndrome (BBS) (Asper Biotech (www.asperbio.com)) [11]. Next generation sequencing technology and target capture offers new efficient and cost effective means for identifying *ALMS1* mutations and will be useful for carrier testing [12].

The primary ocular differential diagnoses, due to the severe retinal dystrophy and visual impairment during the first months of life, include cone dystrophies such as Leber’s congenital amaurosis (LCA), and achromatopsia. Often an initial diagnosis of LCA is revised as further characteristic metabolic phenotypes emerge. In addition, infants with DCM and congestive heart failure (CHF) commonly receive a diagnosis of idiopathic infantile DCM, infectious myocarditis, immune abnormalities, or mitochondrial dysfunction. The phenotypic characteristics of Alström syndrome also resemble Bardet-Biedl syndrome (BBS), a multi-system disease that has the closest clinical similarity to Alström syndrome. Other differential diagnoses include Wolfram, Cohen, Biemond II and Usher syndromes. 

Despite its rarity, there is considerable interest in understanding the molecular basis and biochemical pathways of Alström syndrome as a monogenic model for metabolic syndrome with obesity, insulin resistance, diabetes mellitus, hypertriglyceridemia and hypertension as common features. Additionally, other clinical features, such as retinal degeneration, sensorineural hearing loss, cardiomyopathy, and hepatic and renal failure, are common in the general population. 

The normal function of the ALMS protein and the reasons that its disruption leads to the diverse phenotypes remain unclear, although roles in ciliary function, intracellular trafficking and, most recently, adipocyte differentiation have been reported [13-17]. Identification of monogenic forms of obesity, such as Alström syndrome, and continued research into rare obesity-related disorders can expand our understanding of the mechanisms of energy homeostasis, body weight regulation, and endocrine function.

## ALSTRÖM SYNDROME CLINICAL OVERVIEW

### Neurosensory Deficits

Nystagmus and extreme photophobia or light sensitivity characterizes the ocular presentation of infants and very young children with Alström syndrome. Visual dysfunction is usually demonstrated within a few weeks after birth, with only weak activity in cone electroretinography (ERGs) at 6 months of age or less. By 2.5 years, full-field ERG findings often include severely reduced or extinguished responses with a typical pattern of cone dystrophy and subnormal rod activity. Visual evoked potential (VEP) amplitudes are severely reduced. Rods are preserved initially, but by 5 years, rods are beginning to be destroyed and responses can become unrecordable. Complete blindness usually occurs in the second decade [18]. However, phenotypic variation in age of onset and rate of progression is observed [8]. Bilateral subcapsular cataracts are common, and their removal can temporarily improve light perception. Retinal pathological changes developing in childhood also include attenuation of retinal vessels, optic disc pallor, and increasingly significant retinal pigmentary epithelial (RPE) atrophy. Histological studies demonstrate asteroid hyalosis, optic disc drusen, and bone spicules [8,18-20]. Optical coherence tomography (OCT) imaging in a 5 year old child showed thinning of the macula and an early arrest of macular development with immature retinal structural organization [9]. No therapy exists for the progressive vision loss, but early intervention with low vision aids and magnifiers, mobility training, and Braille should be undertaken in children in expectation of future blindness. 

Eighty-nine percent of individuals develop slowly progressive bilateral sensorineural hearing loss in the first decade that usually progresses to the moderate –to- severe range. However, age of onset and severity is variable and can develop anytime from onset in infancy to adulthood [21,22]. Further, chronic and acute otitis media in childhood often exacerbates the sensorineural deficits with a component of conductive hearing loss [4]. Myringotomy can be of benefit in some individuals with chronic otitis media. Cochlear implantation also has been successful, but surgery complications are known to occur in this rare syndrome [23].

### Obesity

**Fig. (1):**
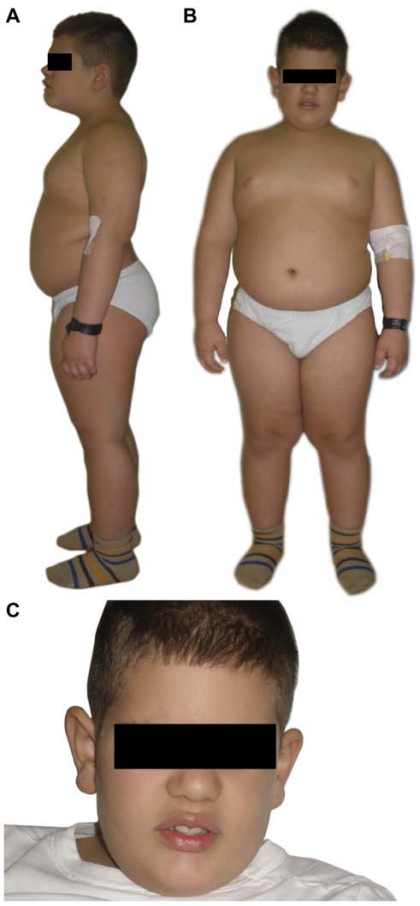
**A, B**. Clinical pictures of a male with Alström syndrome at the age of 6 years, 8 months presenting characteristic truncal obesity. **C**. Note characteristic face and prominent ears.

Obesity is an early and consistent feature observed in most children with Alström syndrome [3, 4]. However, birth weight and body mass index (BMI kg/m^2^) are in the normal range during the first few months of life. Significant and rapid weight gain begins within the first or second year and remains a medically significant problem, particularly in childhood (Fig. **1**). The distribution of adipose tissue is widespread but predominantly distributed subcutaneously and viscerally. Dual energy x-ray absorptiometry (DEXA) scans have estimated total body fat in the top 25th centile [24,25]. BMI for males and females range between 21 and 53 [4]. BMI tends to normalize in older individuals, whereas insulin resistance continues to increase [24, 26]. The moderation of BMI with age does not correlate with the onset of renal or heart failure and T2DM [24].

Decreased levels of physical activity, often exacerbated by dual neurosensory losses, have been observed in Alström syndrome, but no formal metabolic studies have been undertaken. Although childhood hyperphagia has been proposed as a possible issue contributing to obesity, the evidence remains anecdotal [4, 27, 28]. A combination of disordered appetite regulation and decreased physical activity could, therefore, contribute to the development of obesity in this disorder. 

Leptin levels are elevated in Alström syndrome and correlate with body weight [29]. However, mild elevations in leptin levels normalized to BMI, suggest leptin resistance [30]. No formal trials to manage obesity with appetite suppressors or lipase inhibitor therapies have been reported. 

### Type 2 Diabetes Mellitus 

Severe insulin resistance, hyperinsulinemia, and impaired glucose tolerance often present in very early childhood and are frequently accompanied by acanthosis nigricans. Even when matched for pubertal stage and body composition, individuals with Alström syndrome are much more severely insulin resistant than controls [24]. T2DM develops in childhood, adolescence, or adulthood, with a mean age of onset at 16 years [4]. 

Reduced carbohydrate intake may prove more effective than fat restriction for control of hyperglycemia and hyperinsulinemia in a subset of individuals with Alström syndrome [31]. Lee and colleagues [28] described moderation of hyperinsulinemia with strict caloric restriction in a young child with Alström syndrome [32]. Metformin and dipeptidyl peptidase 4 (DPP4) inhibitors have been effective in some individuals [26,31]; however, the hyperglycemia can be intractable thus requiring insulin to achieve glycemic control [33]. Unlike in the general population, the onset of T2DM appears unrelated to the degree of obesity, and protection from clinical peripheral sensory neuropathy has been suggested [34]. 

### Dyslipidemia

Children with Alström syndrome often have high lipid levels at an early age. Hypertriglyceridemia is variable and not always accompanied by hypercholesterolemia. There is no apparent correlation between high triglycerides and insulin resistance, hyperinsulinemia, or obesity [35]. The hypertriglyceridemia can be sufficiently severe to cause pancreatitis [36, 37]. An increased risk for cardiovascular disease has been suggested due to the complications of obesity, severe insulin resistance, T2DM, and renal impairment from an early age [38]. However, MRI imaging studies have shown that cardiac and renal failures found in Alström syndrome are not associated with vascular disease in the coronary artery [39].

### Cardiomyopathy 

DCM and CHF manifest in approximately two-thirds of individuals with Alström syndrome at some stage during their lives and are major causes of morbidity and mortality. Individuals are at risk of sudden abrupt onset of CHF at any age, but often during infancy with onset in the first weeks of life prior to the appearance of other clinical features of Alström syndrome [3, 40, 41]. Many of these infants survive with apparent recovery of cardiac function following treatment. However, after a variable interval of normal to low-normal cardiac function, CHF can suddenly recur in adolescence or adulthood with involvement of both ventricles, rapid progression, and a poor clinical prognosis. Another subset of individuals develop adult or adolescent onset CHF, which appears to be a fibrotic process causing myocardial hypertrophy and dilation with restrictive impairment of both ventricles [39, 42]. Significantly decreased systolic wall motion velocities and amplified aortic systolic pressure are indicative of central arterial stiffening that may contribute to the development of cardiomyopathy [43, 44]. Heart transplantation is problematic, with a poor success rate because multiple organs are compromised in this syndrome. However, successful cardiac transplantation has been reported [45]. 

### Hepatic Pathology

There is extensive phenotypic variation in the slowly progressive hepatic dysfunction in Alström syndrome, which begins with clinically silent elevation of transaminases, and steatosis [46-48]. The initial presentation is usually steatosis and hepatosplenomegaly followed by fibrotic and inflammatory processes with lymphocytic infiltration in the portal and parenchymal areas. Hepatocellular adenoma with pericellular fibrosis has been described [49]. In the final course of hepatic disease, there is significant fibrosis, cirrhosis, portal hypertension, esophageal varicies, encephalopathy, with upper GI hemorrhage leading to death. Inflammatory changes resulting in fibrosis do not appear to be autoimmune related because antinuclear antibodies and other typical markers of autoimmune hepatitis are negative. End stage liver disease is the cause of death in about 10% of individuals [4]. 

### Renal Disease

Manifestations of progressive renal impairment include varying degrees of glomerular disease with reduced glomerular filtration rate and albuminuria. Decreased urine-concentrating capacity, hypertension, renal tubular acidosis, polyuria and polydipsia develop over time. Nephrocalcinosis has been described [50] along with histopathologic focal glomerulosclerosis and interstitial renal fibrosis [4,51]. 

Lower urinary tract dysfunction, recurrent infections, vesicoureteral reflux, urethral stenosis, and detrusor instability have been reported [4, 35]. Urologic dysfunction may require indwelling catheterization. End-stage renal disease (ESRD) can occur as early as the late teens and is a major cause of morbidity. Renal and renal-pancreas transplantation in affected individuals has been successful, although obesity and other compromised organs can render the individual unsuitable for the procedure [52].

### Hypogonadism

Hyper- or hypogonadotropic hypogonadism is seen in both males and females but is more frequent in males. Low-normal levels of testosterone and elevated gonadotropins are suggestive of a primary gonadal failure [53,54]. Males have small external genitalia and testicular atrophy with obliterating fibrosis of seminiferous tubules [4]; however, sporadic spermatozoa can be found in the seminal fluid. Secondary sex characteristics are normal.

Hypogonadism may not be apparent in females until puberty when delay in onset of secondary sex characteristics and menarche become evident. Affected females may have hyperandrogenism, hirsutism, and alopecia, which are probably related to insulin resistance [10]. Pathological examination reveals cystic ovaries with dense fibrotic changes, minimal or absent primary or secondary follicles and no corpora lutea [4]. Abnormal breast development, precocious puberty, endometriosis, irregular menses or amenorrhea has been reported. As children approach puberty, gonadotropin and sex hormone levels should be monitored to determine if hormone replacement therapy is indicated. Metformin, progesterones, or estrogen-progesterone medications have ameliorated cycle regulation in females. No individuals with Alström syndrome are known to have reproduced. 

### Endocrine Disturbances

Prior to puberty, children typically have a height above the 50th percentile, with rapid growth and a bone age advanced by 1-3 years [4]. However, the early growth velocity declines as the child matures, and most adolescents and adults have a final short stature. Abnormalities of the insulin-like growth factors (IGFs) system and growth hormone deficiency have been reported [29,55-58]. Although pituitary dysfunction might explain, at least in part, short stature in Alström syndrome, very few studies have specifically addressed this and speculation about the possible effects of growth hormone replacement therapy remains controversial [56-59]. Secondary and sub-clinical hypothyroidism have been reported in a subset of individuals, suggestive of a hypothalamic defect [4,27,35]. No alterations of prolactin or pituitary-adrenal axes have been reported to date.

### Respiratory Illness

Chronic respiratory tract infections beginning in early childhood, leading to chronic bronchitis, asthma, and chronic rhinosinusitis is common, particularly in children. Pulmonary problems range in severity from frequent bronchial infections to chronic obstructive pulmonary disease (COPD) and acute respiratory distress syndrome (ARDS). Pulmonary hypertension is common and histopathological studies have shown early morphological changes indicating an inflammatory process in the small airways. Severe interstitial, obliterating fibrosis has been reported, [4] with some individuals not able to maintain adequate oxygen saturation and requiring continuous positive air pressure (CPAP). Blood oxygen saturation can plummet rapidly, particularly during or following surgical procedures [23,60].

### Neurologic and Developmental Abnormalities

Most individuals with Alström syndrome demonstrate normal intelligence, although mild to moderate delay in reaching major developmental milestones including gross motor and fine motor skills and intellectual development have been reported [40,61]. The delays in early milestones could be due to sensory deficits although autistic spectrum behaviors and seizure activity have been reported [4]. Balance disturbances have been observed [62,63] but without ataxia or impaired coordination. Some individuals lack facial movement sometimes associated with speech difficulty [62]. Cerebellar anomalies have been noted [64]. Although there are some individuals with Alström syndrome that seem to be totally free of psychological issues, major depression, obsessive-compulsive behavior, and psychotic behavior have been noted, particularly in adults and there are a range of classic medications that have been administered.  It remains to be investigated whether psychological problems are primary or secondary to vision and hearing impairment and general ill health.

### Other Abnormalities

**Fig. (2):**
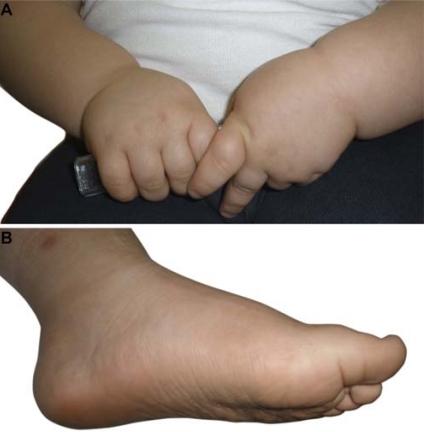
**A**. Close-up of broad hands with stubby fingers and brachydactyly from a 3 year, 3 month old child with Alström syndrome. **B**. Pes planus typically seen in Alström syndrome.

Distinctive facial characteristics are reported in Alström syndrome such as deep-set eyes with a rounded face, hyperostosis frontalis interna, thick ears, premature frontal balding, and thin hair (Fig. **1**). Most children have characteristic wide, thick and flat feet, and short stubby fingers and toes with brachydactyly, but with no polydactyly or syndactyly [4] (Fig. **2**). Scoliosis and kyphosis are frequent and variable in severity and compound cardiac problems by restricting lung function. Individuals may present with chronic abdominal pain, abdominal distension or constipation which may resolve spontaneously. Cecal volvulus has been reported [60].

Dental anomalies such as absent, mislocated or extra teeth are reported and can include gingivitis and light yellow-brown discolored enamel bands on the anterior teeth. Histological examination of the gingiva has revealed irregular thickness of the basal lamina and delamination of the myelin sheath [65]. 

## GENETICS

Alström syndrome is caused by mutations in the *ALMS1* gene, located on chromosome 2p13, and is inherited as an autosomal recessive disorder. Heterozygous carriers are asymptomatic [27]. In contrast to BBS, cases of tri-allelic inheritance have not been reported. 

### *ALMS1* Gene Structure and Expression

Originally described by Collin *et al*. [5] and Hearn *et al. *[6], *ALMS1* is composed of 23 exons. There is strong evidence for several alternatively spliced transcripts. However, the exonic structure of the individual cDNAs has not yet been conclusively identified [5]. Purvis *et al*. [66] characterized the transcription start site (TSS) of the *ALMS1 *gene and examined several tissues which showed multiple, tissue-specific TSSs spanning a 70bp region. *In silico* analysis revealed putative transcription factor binding sites including X-box and three GC-box like elements. Deletion constructs lacking these binding sites have resulted in reduced promoter activity. Since X-box and GC boxes are target sites for RFX and Sp/KLF transcription factors, respectively, targeted mutations were used to show that they disrupted the binding of RFX members and Sp1. Reporter assays showed that Sp1 and RFX proteins can affect the transcription of *ALMS1*. ChIP analysis confirmed the binding of RFX1 and 2 to ALMS1 *in vivo*. RNAi experiments validated the involvement of RFX factors in the upregulation of *ALMS1* specifically following serum starvation [66]. Interestingly, RFX transcription factors regulate genes involved in ciliary assembly supporting a role for ALMS1 in the function/maintenance of cilia.

*ALMS1* is expressed in most tissues affected in Alström syndrome, including the organ of Corti, retinal photoreceptors, renal tubules, liver, and pancreatic islets [5,14,67]. In the brain, *ALMS1* is found widely expressed in most regions including the hypothalamus (http://www.brain-map.org). Several splice variants of *ALMS1* have been described encoding isoforms of the protein [5,13,14]. Some protein isoforms show distinct intracellular localization since antibodies directed against the N-terminal part of the protein label the centrosomes in cells, antibodies against the C-terminus stain the cleavage furrow of dividing cells. Different ALMS1 isoforms may perform different functions in the cell.

The longest *ALMS1* transcript potentially encodes a 461 kDa protein of 4169 amino acids. Exon 1 contains a tract of glutamic acid residues (aa 13–29) consisting of a (GAG)NGAA(GAG)3 repeat, followed by a stretch of seven alanine residues (aa 30−36) [5, 6]. Exon 8, a large 6-kb exon, contains a large tandem repeat domain encoding 34 imperfect repeats of 45–50 amino acids. This domain constitutes 40% of the protein [6]. Potential nuclear localization signals (aa 3805-3830; aa 3937-3954) are found near the carboxy terminus. The carboxy end of the protein represents an evolutionarily conserved motif, termed the ALMS motif, which shares sequence similarity with two other centrosomal proteins, C10orf90 and KIAA1731 [6,68]. Although deletion constructs containing the ALMS motif localized to the centrosome, Knorz *et al*. [68] found that this motif was not critical for centrosome targeting.

At least one isoform of ALMS1 prominently localizes to the centrosomes and basal bodies of ciliated cells. Centrosomes are the microtubule organizing centers of the cell that orchestrate essential aspects of cell division and signaling pathways such as Sonic Hedgehog and WNT, through the primary cilium, respectively [69,70]. Knorz *et al*. [68] showed that ALMS1 interacts with and perhaps anchors the centrosome cohesion protein, CNAP1, to the distal region of basal bodies. Interestingly, CNAP1 has been shown to be important for the linkage between the two basal bodies formed by rootletin fibers [71]. 

### Genetic Variation

**Fig. (3):**
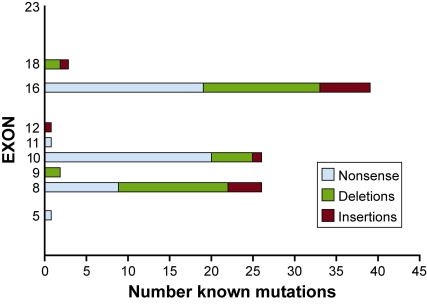
Summary of known mutations in *ALMS1* gene. This includes 94 published alterations [5-11,24,51,72,73] and 15 additional unpublished mutations.

To date, 109 different mutations in *ALMS1* have been identified in individuals with Alström syndrome and the majority are nonsense and frameshift (insertions or deletions) that result in premature termination codons [5-8,10,11,72-73]. A preponderance of disease causing mutations described thus far are located in the coding regions of exons 8, 10 and 16 (Fig. **3**) with mutations in exon 16, accounting for 36% of the total mutational load in Alström syndrome [11,24,73]. Common mutations identified include c.11449C>T; p.Q3817X and c.11313_11316del TAGA; p.D3771fs, and c.10483C>T; p.Q3495X, each identified in 10 unrelated families. Founder effects have been observed in certain ethnic backgrounds: a 19 basepair insertion*,* c.10539_10557ins(n)19; p.H3512fs, has been observed in families of Acadian origin, a 10775delC insertion in UK families, and an IVS8 + 895del1444 deletion in Pakistani families [5,7,41]. The lack of disease causing mutations in the 5’ half of the coding region of *ALMS1* (exons 1 through 7) prompts the speculation that mutations in this region are embryonic lethal. This is supported by the finding in cell culture that knockdown with siRNA targeted to the 5’-end prevents formation of primary cilia [16].

A total of 222 unique SNPs in the human *ALMS1 *gene are listed in the public SNP database which is available at http://uswest.ensembl.org/Homo_sapiens/Gene/Variation_Gene/Table?db=core;g=ENSG00000116127;r=2:73612886-73837920;t=ENST00000264448, March, 2011. SNPs located within the coding region of *ALMS1* show a somewhat similar distribution as that observed with disease causing mutations with a trend for higher SNP density towards the 3’ half of the coding region. Among these, 111 SNPs currently listed in the ENSEMBL database [ENSEMBL, http://www.ensembl.org] lead to non-synonymous coding changes (ascertained in several population surveys). Among these variations is a stop codon in exon 8 (7126G>T, rs34997036) with an allele frequency of 0.014. Since known *ALMS1* mutations have been identified close by, such a change can be expected to cause the disease in either a homozygous state or as a compound heterozygote together with another *ALMS1* mutation. In addition, in the general population there are two insertions causing a frameshift and predicted premature termination (2048insC, rs344442050; 11983insT, rs34465329), which might be expected to be disease alleles. Assuming the high allele frequencies are not artifacts of the yet low coverage of the general population (~100 individuals), one might expect the disease to be more prevalent. This is not entirely implausible considering the high variability in phenotype and the results from murine studies indicating that most Alström syndrome phenotypes are strongly modified by the genetic background which may result in a less obvious clinical phenotype.

The *ALMS1* gene shows an unusual population structure and evolutionary history. It is one of several genes that shows strong positive recent selection, on par with lactase persistence and resistance to malaria, and among the strongest selection estimated for humans [74]. The prevalence of specific haplogroups in African, European and Asian populations was best explained by selection of preexisting variation in Eurasian populations about 15,000 years ago. It is currently unclear what the selective pressure acting on *ALMS1* might be, although Scheinfeld *et al*. [74] point out that several other regions of selection in the human genome have been associated with carbohydrate metabolism. Interestingly, SNP rs7598660 was found to be weakly associated with several insulin and glucose related traits, such as 2 hour insulin levels, with the ancestral allele linked to higher insulin levels or greater insulin resistance. Although it is unlikely that this association is causally related to the selective sweep in Eurasians, it is possible that a variation in *ALMS1* contributing to insulin resistance was carried on the selected haplotype, and may today contribute to the higher incidence of insulin resistance in Americans of African ancestry compared to those with European ancestry [74]. 

### Association Studies

Correlation studies have examined whether some of the phenotypic variation in Alström syndrome individuals could be attributed to allele specificity. In small-scale studies (≤12 Alström syndrome kindreds), no correlations were found between mutation positions and the occurrence of specific clinical features such as dilated cardiomyopathy [24,41]. Interestingly, in a larger phenotype and genotype analysis, Marshall* et al*. [7] found a suggestive correlation for mutations in exon 8 and normal renal function.

Common variations in loci tightly linked to *ALMS1* have been shown to be associated with kidney disease [75]. In a genome-wide association study of cohorts from within the CKDGen consortium, an association was found for a SNP residing in close proximity to *ALMS1*, rs13538, and renal filtration rate. In 22,982 Caucasian individuals, the association was confirmed for renal marker, cystatin C (eGFRcys) [76]. Chambers *et al*. [77] also showed an association with SNP rs10206899 (lies in close proximity to *ALMS1*) and serum creatinine levels. In small population-based association studies in the UK and The Netherlands, no associations were found among common variants in *ALMS1* and T2DM [78,79]. However, in an extensive population genomic analysis of *ALMS1*, Scheinfeldt *et al*. [74] showed that variations in *ALMS1* may contribute to interindividual differences in metabolic phenotypes. 

## ANIMAL AND CELLULAR MODELS OF ALSTRÖM SYNDROME

Several mouse models for Alström syndrome*, *including gene-trapped, ENU-mutagenized and spontaneous mutations, have been well characterized [13,16,80]. These models recapitulate the features observed in individuals with Alström syndrome and, therefore, have the potential to provide new insights into the mechanisms of many of the fundamental pathologic features of Alström syndrome (Table **1**). In addition, many of the phenotypes observed in the animal models, such as retinal degeneration, obesity, and fibrosis, can be modified by the genetic background, suggesting that the variability seen in the human population may also be, at least in part, due to genetic modifiers.

### Mouse Models

#### Alms1^Gt(XH152)Byg^

The *Alms1* gene-trapped mouse model, *Alms1^GT/GT^*, exhibits most of the classic disease phenotypes found in individuals with Alström syndrome [13]. *Alms1^GT/GT^* mice were not hyperphagic, nor did they display alterations in energy consumption before the increase in body weight and the resulting hyperleptinemia (*unpublished results*). Ultrastructural examination of cilia in *Alms1^GT/GT^* mice showed normal ciliogenesis in renal tubules, nasal epithelial cells, and photoreceptors. In the cochlea, ALMS1 immunolocalizes to the basal bodies of the kinocilium and stereocilia with lesions in the stria vascularis, and abnormalities in the hair cell stereociliary bundles indicative of planar cell polarity defects [67].

#### Alms1^foz^

Another mouse model, *fat aussie *(*foz/foz*), like the gene trap model, exhibits several of the pathologies found in Alström syndrome. *Foz/foz* mice fed a normal chow diet develop simple steatosis, while those fed a high fat diet develop nonalcoholic steatohepatitis [80-82]. Short term therapeutic studies with Wy-14, 643, a peroxisome proliferation activator receptor alpha (PPAR-∝) agonist, have shown that enhancement of fatty acid oxidation reduced steatosis. However, the treatment failed to restore sinusoidal blood flow in *foz/foz *livers [83].

#### Alms1^L2131X^

In the ENU-induced* Alms1^L2131X/L2131X^* mice, collecting renal tubules develop normal primary cilia. However, the cortical tubules show some loss of cilia as the mouse ages. *In vitro* knockdown of the disease allele inhibited ciliogenesis, thereby suggesting some residual function of ALMS1 [16].

### *In Vitro* Studies

#### Mouse Renal Inner Medullary Collecting Duct (mIMCD) Cells

*In vitro* knockdown of the 5’ end of *ALMS1* in mIMCD cells using short interfering RNA (siRNA) resulted in abnormally stunted renal cilia, which were unable to induce calcium flux in response to environmental cues [16]. These results support a role for ALMS1 in ciliogenesis and ciliary function. However, loss of ALMS1 did not alter transcriptional control of ciliary genes. 

#### 3T3L1 Cells

Expression studies of preadipocytes from 3T3L1 cells induced with adipogenic factors showed that *Alms1* expression decreases during the differentiation of preadipocytes to mature adipocytes. However, treatment of preadipocytes with adipogenic factors did not significantly modify *Alms1* expression, suggesting a role of the gene in the early phase of adipogenesis [17]. Moreover, knockdown of *Alms1* in 3T3L1 cells showed impairment in adipocyte differentiation but does not appear to have an effect on cell-autonomous insulin action [84]. These results suggest that partial impairment of adipogenesis in Alström syndrome may contribute to the severity of the associated metabolic phenotype. 

#### Human Primary Fibroblasts

To explore the hypothesis that a defect in adipogenesis and/or insulin action in mature adipocytes leads to relative failure of metabolic homeostasis, dermal fibroblasts derived from Alström syndrome individuals were examined *in vitro*. Insulin receptor mRNA is increased, despite normal early signaling events in response to insulin and normal insulin-dependent glucose uptake [85]. 

## CONCLUSION

Alström syndrome is a complex pleiotropic disorder caused by mutations in *ALMS1*. Systemic characteristics of this disorder include truncal obesity, hyperinsulinemia, and T2DM, acanthosis nigricans, short adult stature, and vision and hearing loss, dilated or restrictive cardiomyopathy (infancy or adolescence/adulthood) with CHF, hypothyroidism and hypogonadism. Death usually occurs due to progressive cardiac, hepatic and renal failure, often associated with pulmonary disease. The cellular and molecular mechanisms underlying the disorder remain to be understood although widespread severe fibrosis may significantly contribute to the disease pathology. Studies in murine and cellular models suggest that defects in ciliogenesis and/or structure, function, or maintenance of cilia underlie the pathogenesis of Alström syndrome [13-16,68]. 

The consequences of centrosome, ciliary, and/or IFT system deficits in the hypothalamic pathways for metabolic abnormalities leading to obesity are not well understood. It has been hypothesized that loss of functioning ALMS1 could impact the cilia on hypothalamic neurons and lead to alteration in behavior and energy homeostasis, through abnormal perception of appetite and satiety cues, such as leptin, resulting in overeating and obesity [86]. In other organ systems such as the cochlea and the proximal renal tubules, planar cell polarity defects, which are often observed in ciliopathies, may play a role. In yet other organs, such as the heart, the role of ALMS1 in the pathology is entirely unclear. However, the knowledge that ALMS1 is a ciliary protein and plays a role in normal centrosome/basal body function and their associated intracellular trafficking events allows new hypotheses to be formulated and to make further progress in understanding and treating Alström syndrome. 

## Figures and Tables

**Table 1:** Murine Models and Human Comparison with Alström Syndrome

	Human *ALMS1*	Mouse Models - *ALMS1* homolog (human)		
		*Alms1^Gt(XH152)Byg^*	*Alms1^foz^* (fat aussie)	*Alms1^L2131X^*
**References**		[13,67]	[80-82]	[16]
**Mutation Type**	Spontaneous; nonsense and frameshift	Gene trap insertion in intron 13	Spontaneous; 11 bp deletion in exon 8	ENU-induced; nonsense mutation in exon 10
**Vision**	Cone-rod dystrophy; photophobia/nystagmus <1 year	Abnormal cone-rod ERG, Slowly progressive photoreceptor degeneration; accumulation of intracellular vesicles; mislocalization of rhodopsin	Not assessed	Impaired rhodopsin transport
**Hearing**	Sensorineural loss in >85%	Abnormal ABR; abnormal stereocilia; loss of OHC & lesions in stria vascularis	Abnormal ABR >1 year	Not assessed
**Obesity**	100% in children, moderates to high normal weight in adults	Onset 8-12 weeks	Onset by 3 months. Males and females hyperphagic	Onset 7-10 weeks
**T2DM**	95% in children over age 15 year	Early hyperinsulinemia; Males are overtly hyperglycemic (>16 weeks); females have elevated glucose levels	Hyperinsulinemia by 60 days; pancreatic islet hyperplasia and islet cysts; Male and female diabetic	Hyperinsulinemia, but majority are normoglycemic
**Lipids**	Hypertriglyceridemia; hypercholesterolemia	Hypercholesterolemia; normal triglycerides	Hypercholesterolemia; normal triglycerides	Hypercholesterolemia and elevated triglycerides
**Cardiac**	Dilated/hypertrophic cardiomyopathy	Unknown	Unknown	Unknown
**Hypo-gonadism**	<80% males; amenorrhea in females	Male hypogonadic, atrophic seminiferous tubules; lack of sperm Females fertile prior to obesity.	Females fertile prior to obesity; males are sterile; progressive germ cell loss; block of development of spermatids; flagellation defects	Males have defective sperm formation
**Renal**	Glomerulosclerosis	Enlarged kidneys; dilation of proximal tubules; proteinurea; aged mice may develop renal cysts	Dilated cortical tubules, and older animals showed loss of cilia from kidney proximal tubules	Dilated cortical tubules; proteinurea; specific loss of cilia from the kidney proximal tubules in aged mice
**Hepatic**	Steatosis, cirrhosis, hepatosplenomegaly, fibrosis	Steatosis, hepatosteatitis; fibrosis	Chow diet: steatosis: High fat diet: hepatosteatitis, inflammation, and fibrosis	Steatosis

## ABBREVIATIONS

ABR: = Auditory brainstem response
ARDS: = Acute respiratory distress syndrome
BBS: = Bardet-Biedl Syndrome
BMI: = Body mass index (kg/m^2^)
CHF: = Congestive heart failure
CNAP1: = Centrosome cohesion protein
COPD: = Chronic obstructive pulmonary disease
CPAP: = Continuous positive air pressure
DCM: = Dilated cardiomyopathy
DEXA: = Dual energy x-ray absorptiometry
ERG: = Electroretinography
ESRD: = End stage renal disease
IGF: = Insulin-like growth factor
IFT: = Intraflagellar transport
LCA: = Leber’s congenital amaurosis
mIMCD: = Mouse renal inner medullary collecting duct cells
OCT: = Optical coherence tomography
OHC: = Outer hair cells
RPE: = Retinal pigment epithelium
SNP: = Single nucleotide polymorphism
T2DM: = Type 2 diabetes mellitus
TF: = Transcription factor
TSS: = Transcription start site
VEP: = Visual evoked potential

## References

[1] Alström CH, Hallgren B, Nilsson LB, Åsander H (1959). Retinal degeneration combined with obesity, diabetes mellitus and neurogenous deafness. A specific syndrome (not hitherto described) distinct from Laurence-Moon-Biedl syndrome. A clinical endocrinological and genetic examination based on a large pedigree. Acta Psychiatr. Neurol. Scand. Suppl.

[2] Marshall JD, Paisey RB, Carey CM, MacDermott S, Pagon RA, Bird TC, Dolan CR, Stephens K (2010). Gene Reviews [Internet], Seattle (WA): University of Washington, Seattle; 1993-2003.

[3] Marshall JD, Beck S, Maffei P, Naggert JK (2007). Alström Syndrome. Eur. J. Hum. Genet.

[4] Marshall JD, Bronson RT, Collin GB, Nordstrom AD, Maffei P, Paisey RB, Carey C, Macdermott S, Russell-Eggitt I, Shea SE, Davis J, Beck S, Shatirishvili G, Mihai CM, Hoeltzenbein M, Pozzan GB, Hopkinson I, Sicolo N, Naggert JK, Nishina PM (2005). New Alström Syndrome phenotypes based on the evaluation of 182 cases. Arch. Intern. Med.

[5] Collin GB, Marshall JD, Ikeda A, So WV, Russell-Eggitt I, Maffei P, Beck S, Boerkoel CF, Sicolo N, Martin M, Nishina PM, Naggert JK (2002). Mutations in *ALMS1* cause obesity, type 2 diabetes and neurosensory degeneration in Alström syndrome. Nat. Genet.

[6] Hearn T, Renforth GL, Spalluto C, Hanley NA, Piper K, Brickwood S, White C, Connolly V, Taylor JFN, Russell-Eggitt I, Bonneau D, Walker M, Wilson DI (2002). Mutation of *ALMS1*, a large gene with a tandem repeat encoding 47 amino acids, causes Alström syndrome. Nat. Genet.

[7] Marshall JD, Hinman EG, Collin GB, Beck S, Cerqueira R, Maffei P, Milan G, Zhang W, Wilson DI, Hearn T, Tavares P,  Vettor R,  Veronese C, Martin M, So WV, Nishina PM, Naggert JK (2007). Spectrum of *ALMS1* variants and evaluation of genotype-phenotype correlations in Alström syndrome. Hum. Mutat.

[8] Malm E, Ponjavic V, Nishina PM, Naggert JK, Hinman EG, Andréasson S, Marshall JD, Möller C (2008). Full-field electroretinography and marked variability in clinical phenotype of Alström syndrome. Arch. Ophthalmol.

[9] Vingolo EM, Salvatore S, Grenga PL, Maffei P, Milan G, Marshall JD (2010). High resolution spectral domain optical coherence tomography images of Alström syndrome. J. Pediatr. Ophthalmol. Strabis.

[10] Kocova M, Sukarova-Angelovska E, Kacarska R, Maffei P, Milan G, Marshall JD (2010). The unique combination of dermatological and ocular phenotypes in Alström syndrome: Severe presentation, early onset, and two novel *ALMS1* mutations. Br. J. Dermatol.

[11] Pereiro I, Hoskins BE, Marshall JD, Collin GB, Naggert JK, Teresa Piñeiro-Gallego T, Oitmaa E, Katsanis N, Valverde D, Beales PL (2011). Arrayed Primer Extension (APEX) technology simplifies mutation detection in Bardet Biedl and Alström Syndrome. Eur. J. Hum. Genet.

[12] Bell CJ, Dinwiddie DL, Miller NA, Hateley SL, Ganusova EE, Mudge J, Langley RJ, Zhang L, Lee CC, Schilkey FD, Sheth V, Woodward JE, Peckham HE, Schroth GP, Kim RW, Kingsmore SF (2011). Carrier testing for severe childhood recessive diseases by next-generation sequencing. Sci. Transl. Med.

[13] Collin GB, Cyr E, Bronson R, Marshall JD, Gifford EJ, Hicks W, Murray SA, Zheng QY, Smith RS, Nishina PM, Naggert JK (2005). Alms1-disrupted mice recapitulate human Alström syndrome. Hum. Mol. Genet.

[14] Hearn T, Spalluto C, Phillips VJ, Renforth GL, Copin N, Hanley NA, Wilson DI (2005). Subcellular localization of ALMS1 supports involvement of centrosome and basal body dysfunction in the pathogenesis of obesity, insulin resistance, and type 2 diabetes. Diabetes.

[15] Anderson JS, Wilkenson CJ, Mayor T, Mortenson P, Nigg EA, Mann M (2003). Proteomic characterization of the human centrosome by protein correlation profiling. Nature.

[16] Li G, Vega R, Nelms K, Gekakis N, Goodnow C, McNamara P, Wu H, Hong N, Glynne R (2007). A role for Alstrom syndrome protein, Alms1, in kidney ciliogenesis and cellular quiescence. PLoS Genet.

[17] Romano S, Milan G, Veronese C, Collin GB, Marshall JD, Centobene C, Favaretto F, Dal Pra C, Scarda A, Leandri S, Naggert JK, Maffei P, Vettor R (2008). Regulation of Alström syndrome gene expression during adipogenesis and its relationship with fat cell insulin sensitivity. Int. J. Mol. Med.

[18] Russell-Eggitt IM, Clayton PT, Coffey R, Kriss A, Taylor DSI, Taylor JFN (1998). Alström syndrome. Report of 22 cases and literature review. Ophthalmol.

[19] Sebag J, Albert DM, Craft JL (1984). The Alström syndrome: Ophthalmic histopathology and renal ultrastructure. Brit. J. Ophthalmol.

[20] Tremblay F, LaRoche RG, Shea SE, Ludman MD (1993). Longitudinal study of the early electroretinographic changes in Alström's Syndrome. Am. J. Ophthalmol.

[21] Welsh LW (2007). Alström syndrome: Progressive deafness and blindness. Ann. Otol. Rhinol. Laryngol.

[22] Paisey RB, Carey CM, Parkinson MJ, Parkinson C, Cole MD (2000). Alström syndrome-the case for secondary prevention. Diabet. Res. Clin. Pract.

[23] Florentzson R, Hallén K, Möller C (2010). Alström syndrome and cochlear implantation. The first ? clinical experience.

[24] Minton JA, Owen KR, Ricketts CJ, Crabtree N, Shaikh G, Ehtisham S, Porter JR, Carey C, Hodge D, Paisey R, Walker M, Barrett TG (2006). Syndromic obesity and diabetes: changes in body composition with age and mutation analysis of ALMS1 in 12 United Kingdom kindreds with Alström syndrome. J. Clin. Endocrinol. Metab.

[25] Paisey RB, Hodge D, Williams K (2008). Body fat distribution, serum glucose, lipid and insulin response to meals in Alström syndrome. J. Hum. Nutr. Diet.

[26] Paisey RB (2009). New insights and therapies for the metabolic consequences of Alström syndrome. Curr. Opin. Lipidol.

[27] Marshall JD, Ludman MD, Shea SE, Salisbury SR, Willi SM, LaRoche RG, Nishina PM (1997). Genealogy, natural history, and phenotypic features of Alström syndrome in a large Acadian kindred and three unrelated families. Am. J. Med. Genet.

[28] Maffei P, Munno V, Marshall JD, Scandellari C, Sicolo N (2002). The Alström syndrome: is it a rare or unknown disease?. Ann. Ital. Med. Int.

[29] Maffei P, Boschetti M, Marshall JD, Paisey RB, Beck S, Resmini E, Collin GB, Naggert JK, Milan G, Vettor R, Minuto F, Sicolo N, Barreca A (2007). Characterization of the IGF system in 15 patients with Alström syndrome. Clin. Endocrinol.

[30] Hung Y-J, Jeng C, Pei D, Chou P-I, Wu D-A (2001). Alström Syndrome in Two Siblings. J. Formos. Med. Assoc.

[31] Paisey RB, Hodge D, Bower L (2008). Weight and glycaemic responses to 6 months Exenatide treatment in 9 Alström syndrome subjects with type 2 diabetes. Abstract: 44th EASD Meeting, Rome, Italy.

[32] Lee N-C, Marshall JD, Collin GB, Naggert JK , Chien Y-H, Tsai W-Y, Hwu W-L (2009). Caloric restriction in Alström syndrome prevents hyperinsulinemia – A case report. Am. J. Med. Genet. A.

[33] Satman I, Yilmaz MT, Gürsoy N, Karsidag K, Dinççag N, Ovali T, Karadeniz S, Uysal V, Bugra Z, Ökten A, Devrim S (2002). Evaluation of insulin resistant diabetes mellitus in Alström syndrome: a long-term prospective follow-up of three siblings. Diabet. Res. Clin. Pract.

[34] Paisey RB, Paisey RM, Thompson MP, Bower L, Maffei P, Shield JPH, Barnett S, Marshall JD (2009). Protection from clinical peripheral sensory neuropathy in Alström Syndrome in contrast to early-onset Type 2 Diabetes. Diabet. Care.

[35] Charles SJ, Moore AT, Yates JRW, Green T, Clark P (1990). Alstrom's syndrome: further evidence of autosomal recessive inheritance and endocrinological dysfunction. J. Med. Genet.

[36] Paisey RB, Carey CM, Bower L, Marshall J, Taylor P, Maffei P, Mansell P (2004). Hypertriglyceridaemia in Alstrom's syndrome: causes and associations in 37 cases. Clin. Endocrinol.

[37] Wu WC, Chen SC, Dia CY, Yu ML, Hsieh MY, Lin ZY, Wang LY, Tsai JF, Chang WY, Chuang WL (2003). Alström syndrome with acute pancreatitis: A case report. Kaohsiung J. Med. Sci.

[38] Atabek ME, Sinha S.K (2007, 2008). Re: "Effect of metformin and rosiglitazone in a prepubertal boy with Alström syndrome". JPEM, J. Pediatr. Endocrinol. Metab.

[39] Loudon M, Bellenger N, Carey C, Paisey R (2009). Cardiac magnetic resonance imaging in Alstrom syndrome. Orphan. J. Rare Dis.

[40] Michaud JL, Héon E, Guilbert F, Weill J, Puech B, Benson L, Smallhorn J, Shuman CT, Buncic JR, Levin AV, Weksberg R, Brevière GM (1996). Natural history of Alström syndrome in early childhood: Onset with dilated cardiomyopathy. J. Pediatr.

[41] Bond J, Flintoff K, Higgins J, Scott S, Bennet C, Parsons J, Mannon J, Jafri H, Rashid Y, Barrow M, Trembath R, Woodruff G, Rossa E, Lynch S, Sheilds J, Newbury-Ecob R, Falconer A, Holland P, Cockburn D, Karbani G, Malik S, Ahmed M, Roberts E, Taylor G, Woods CG (2005). The importance of seeking ALMS1 mutations in infants with dilated cardiomyopathy. J. Med. Genet.

[42] Makaryus A, Zubrow M, Marshall J, Gillam L, Mangion J (2007). Cardiac manifestations of Alström syndrome: echocardiographic findings. J. Am. Soc. Echocardiogr.

[43] Toulany A, Shea S, Warren AE (2006). Doppler tissue, strain, and strain rate imaging in pediatric patients with Alström syndrome: Are There Regional Functional Abnormalities?. J. Am. Soc. Echocardiogr.

[44] Smith JC, McDonnell B, Retallick C, McEniery C, Carey C, Davies JS, Barrett T, Cockcroft JR, Paisey R (2007). Is arterial stiffening in Alström Syndrome linked to the development of cardiomyopathy?. Eur. J. Clin. Invest.

[45] Goerler H, Warnecke G, Winterhalter M, Müller C, Ballmann M, Wessel A, Haverich A, Strüber M, Simon A (2007). Heart-lung transplantation in a 14-year-old boy with Alström syndrome. J. Heart Lung Transplant.

[46] Connolly MB, Jan JE, Couch RM, Wong LTK, Dimmick JE, Rigg JM (1990). Hepatic dysfunction in Alström disease. Am. J. Med. Genet.

[47] Awazu M, Tanaka T, Sato S, Anzo M, Higuchi M, Yamazaki K, Matsuo N (1997). Hepatic dysfunction in two sibs with Alström syndrome: Case report and review of the literature. Am. J. Med. Genet.

[48] Quiros-Tejeira R E, Vargas J, Ament ME (2001). Early-onset liver disease complicated with acute liver failure in Alström syndrome. Am. J. Med. Genet.

[49] Morgan J, Sadler MA, Siegel S (2008). US, CT, and MR imaging of hepatic masses in Alström syndrome: a case report. Clin. Imaging.

[50] Mastrapasqua SC (2008). Sindrome de Alstrom. Primer caso descripto en Argentina. Rev. Nefrolog. Dil. Traspl.

[51] Izzi C, Maffei P, Milan G, Tardanico R, Foini P, Marshall JD, Scolari F (2011). The Case | Familial occurrence of retinitis pigmentosa, deafness, and renal involvement. Kidney Int.

[52] Richardson D, Shires M, Davison AM (2001). Renal diagnosis without renal biopsy. Nephritis and sensorineural deafness. Nephrol. Dial. Transplant.

[53] Warren SE, Schnitt SJ, Bauman AJ, Gianelly RE, Landsberg L, Baim DS (1987). Late onset dilated cardiomyopathy in a unique familial syndrome of hypogonadism and metabolic abnormalities. Am. Heart J.

[54] Weinstein RL, Kliman B, Scully RE (1969). Familial syndrome of primary testicular insufficiency with normal virilization, blindness, deafness and metabolic abnormalities. New Engl. J. Med.

[55] Maffei P, Munno V, Marshall JD, Milanesi A, Martini C, De Carlo E, Mioni R, Pontoni E, Menegazzo C, Sicolo N (2000). GH and IGF-I Axis in Alström Syndrome. J. Endocrinol. Invest.

[56] Maffei P, Marshall JD, Vettor R, Collin GB, Sicolo N, Naggert JK, Harsch I (2005). Alström syndrome. The Various Types and Treatments of Obesity.

[57] Alter CA, Moshang TJ (1993). Growth hormone deficiency in two siblings with Alström syndrome. Am. J. Dis. Child.

[58] Mihai CM, Catrinoiu D, Toringhibel M, Stoicescu RM, Negreanu-Pirjol T, Hancu A (2009). Impaired IGF1-GH axis and new therapeutic options in Alström Syndrome patients: a case series. Cases J.

[59] Tai TS, Lin SY, Sheu WH (2003). Metabolic effects of growth hormone therapy in an Alstrom syndrome patient. Horm. Res.

[60] Khoo EY, Risley J, Zaitoun AM, El-Sheikh M, Paisey RB, Acheson AG, Mansell P (2009). Alström syndrome and cecal volvulus in 2 siblings. Am. J. Med. Sci.

[61] Hamamy H, Barham M, Alkhawaldeh AE, Cockburn D, Snowden H, Ajlouni K (2006). Alström syndrome in four sibs from Northern Jordan. Ann. Saudi Med.

[62] Möller C (2005). Alström syndrome. The National Swedish board of Health and Welfare.

[63] Möller C, Newton W (2009). Vestibular testing in children. Pediatric Audiological Medicine.

[64] Yilmaz C, Çaksen H, Yilmaz N, Güven AS, Arslan D, Cesur Y (2006). Alstrom Syndrome associated with cerebral involvement: An unusual presentation. Eur. J. Gen. Med.

[65] Koray F, Corter C, Benderli Y, Satman I, Yilmaz T, Dinççag N, Karsidag K (2001). Alström syndrome: a case report. J. Oral Sci.

[66] Purvis TL, Hearn T, Spalluto C, Knorz VJ, Hanley KP, Sanchez-Elsner, Hanley NA, Wilson DI (2010). Transcriptional regulation of the Alström syndrome gene *ALMS1* by members of the RFX family and Sp1. Gene.

[67] Jagger D, Collin G, Kelly J, Towers E, Nevill G, Longo-Guess C, Benson J, Halsey K, Dolan D, Marshall J, Naggert J, Forge A (2011). Alström Syndrome protein ALMS1 localizes to basal bodies of cochlear hair cells and regulates cilium-dependent planar cell polarity. Hum. Mol. Genet.

[68] Knorz VJ, Spalluto C, Lessard M, Purvis TL, Adigun FF, Collin GB, Hanley NA, Wilson DI, Hearn T (2010). Centriolar association of ALMS1 and likely centrosomal functions of the ALMS motif- containing proteins C10orf90 and KIAA1731. Mol. Biol. Cell.

[69] Nigg EA, Raff JW (2009). Centrioles, centrosomes, and cilia in health and disease. Cell.

[70] Wallingford JB, Mitchell B (2011). Strange as it may seem: the many links between Wnt signaling, planar cell polarity, and cilia. Genes Dev.

[71] Yang J, Adamian M, Li T (2006). Rootletin interacts with C-Nap1 and may function as a physical linker between the pair of centrioles/basal bodies in cells. Mol. Biol. Cell.

[72] Aldahmesh MA, Abu-Safieh L, Khan AO, Al-Hassnan ZN, Shaheen R, Rajab M, Monies D, Meyer BF, Alkuraya FS (2009). Allelic heterogeneity in inbred populations: The Saudi experience with Alström syndrome as an illustrative example. Am. J. Med. Genet. A.

[73] Joy T, Cao H, Black G, Malik R, Charlton-Menys V, Hegele RA, Durrington PN (2002). Alström syndrome (OMIM 203800): a case report and literature review. Orphan. J. Rare Dis.

[74] Scheinfeldt LB, Biswas S, Madeoy J, Connelly CF, Schadt EE, Akey JM (2009). Population genomic analysis of ALMS1 in humans reveals a surprisingly complex evolutionary history. Mol. Biol. Evol.

[75] International HapMap Consortium Frazer KA, Ballinger DG, Cox DR, Hinds DA, Stuve LL, Gibbs RA, Belmont JW, Boudreau A, Hardenbol P, Leal SM, Pasternak S, Wheeler DA, Willis TD, Yu F, Yang H, Zeng C, Gao Y, Hu H, Hu W, Li C, Lin W, Liu S, Pan H, Tang X, Wang J, Wang W, Yu J, Zhang B, Zhang Q, Zhao H, Zhao H, Zhou J, Gabriel SB, Barry R, Blumenstiel B, Camargo A, Defelice M, Faggart M, Goyette M, Gupta S, Moore J, Nguyen H, Onofrio RC, Parkin M, Roy J, Stahl E, Winchester E, Ziaugra L, Altshuler D, Shen Y, Yao Z, Huang W, Chu X, He Y, Jin L, Liu Y, Shen Y, Sun W, Wang H, Wang Y, Wang Y, Xiong X, Xu L, Waye MM, Tsui SK, Xue H, Wong JT, Galver LM, Fan JB, Gunderson K, Murray SS, Oliphant AR, Chee MS, Montpetit A, Chagnon F, Ferretti V, Leboeuf M, Olivier JF, Phillips MS, Roumy S, Sallée C, Verner A, Hudson TJ, Kwok PY, Cai D, Koboldt DC, Miller RD, Pawlikowska L, Taillon-Miller P, Xiao M, Tsui LC, Mak W, Song YQ, Tam PK, Nakamura Y, Kawaguchi T, Kitamoto T, Morizono T, Nagashima A, Ohnishi Y, Sekine A, Tanaka T, Tsunoda T, Deloukas P, Bird CP, Delgado M, Dermitzakis ET, Gwilliam R, Hunt S, Morrison J, Powell D, Stranger BE, Whittaker P, Bentley DR, Daly MJ, de Bakker PI, Barrett J, Chretien YR, Maller J, McCarroll S, Patterson N, Pe'er I, Price A, Purcell S, Richter DJ, Sabeti P, Saxena R, Schaffner SF, Sham PC, Varilly P, Altshuler D, Stein LD, .E. Cutler , .J. Kashuk, C.S; Lin S, Abecasis GR, Guan W, Li Y, Munro HM, Qin ZS, Thomas DJ, McVean G, Auton A, Bottolo L, Cardin N, Eyheramendy S, Freeman C, Marchini J, Myers S, Spencer, Stephens M, Donnelly P, Cardon LR, Clarke G, Evans DM, Morris AP, Weir BS, Tsunoda T, Mullikin JC, Sherry ST, Feolo M, Skol A, Zhang H, Zeng C, Zhao H, Matsuda I, Fukushima Y, Macer DR, Suda E, Rotimi CN, Adebamowo CA, Ajayi I, Aniagwu T, Marshall PA, Nkwodimmah C, Royal CD, Leppert MF, Dixon M, Peiffer A, Qiu R, Kent A, Kato K, Niikawa N, Adewole IF, Knoppers BM, Foster MW, Clayton EW, Watkin J, Gibbs RA, Belmont JW, Muzny D, Nazareth L, Sodergren E, Weinstock GM, Wheeler DA, Yakub I, Gabriel SB, Onofrio RC, Richter DJ, Ziaugra L, Birren BW, Daly MJ, Altshuler D, Wilson RK, Fulton LL, Rogers J, Burton J, Carter NP, Clee CM, Griffiths M, Jones MC, McLay K, Plumb RW, Ross MT, Sims SK, Willey DL, Chen Z, Han H, Kang L, Godbout M, Wallenburg JC, L'Archevêque P, Bellemare G, Saeki K, Wang H, An D, Fu H, Li Q, Wang Z, Wang R, Holden AL, Brooks LD, McEwen JE, Guyer MS, Wang VO, Peterson JL, Shi M, Spiegel J, Sung LM, Zacharia LF, Collins FS, Kennedy K, Jamieson R, Stewart J (2007). A second generation human haplotype map of over 3.1 million SNPs. Nature.

[76] Köttgen A, Pattaro C, Böger CA, Fuchsberger C, Olden M, Glazer NL, Parsa A, Gao X, Yang Q, Smith AV, O'Connell JR, Li M, Schmidt H, Tanaka T, Isaacs A, Ketkar S, Hwang SJ, Johnson AD, Dehghan A, Teumer A, Paré G, Atkinson EJ, Zeller T, Lohman K, Cornelis MC, Probst-Hensch NM, Kronenberg F, Tönjes A, Hayward C, Aspelund T, Eiriksdottir G, Launer LJ, Harris TB, Rampersaud E, Mitchell BD, Arking DE, Boerwinkle E, Struchalin M, Cavalieri M, Singleton A, Giallauria F, Metter J, de Boer IH, Haritunians T, Lumley T, Siscovick D, Psaty BM, Zillikens MC, Oostra BA, Feitosa M, Province M, de Andrade M, Turner ST, Schillert A, Ziegler A, Wild PS, Schnabel RB, Wilde S, Munzel TF, Leak TS, Illig T, Klopp N, Meisinger C, Wichmann HE, Koenig W, Zgaga L, Zemunik T, Kolcic I, Minelli C, Hu FB, Johansson A, Igl W, Zaboli G, Wild SH, Wright AF, Campbell H, Ellinghaus D, Schreiber S, Aulchenko YS, Felix JF, Rivadeneira F, Uitterlinden AG, Hofman A, Imboden M, Nitsch D, Brandstätter A, Kollerits B, Kedenko L, Mägi R, Stumvoll M, Kovacs P, Boban M, Campbell S, Endlich K, Völzke H, Kroemer HK, Nauck M, Völker U, Polasek O, Vitart V, Badola S, Parker AN, Ridker PM, Kardia SL, Blankenberg S, Liu Y, Curhan GC, Franke A, Rochat T, Paulweber B, Prokopenko I, Wang W, Gudnason V, Shuldiner AR, Coresh J, Schmidt R, Ferrucci L, Shlipak MG, van Duijn CM, Borecki I, Krämer BK, Rudan I, Gyllensten U, Wilson JF, Witteman JC, Pramstaller PP, Rettig R, Hastie N, Chasman DI, Kao WH, Heid IM, Fox CS (2010). New loci associated with kidney function and chronic kidney disease. Nat. Genet.

[77] Chambers JC, Zhang W, Lord GM, van der Harst P, Lawlor DA, Sehmi JS, Gale DP, Wass MN, Ahmadi KR, Bakker SJ, Beckmann J, Bilo HJ, Bochud M, Brown MJ, Caulfield MJ, Connell JM, Cook HT, Cotlarciuc I, Davey Smith G, de Silva R, Deng G, Devuyst O, Dikkeschei LD, Dimkovic N, Dockrell M, Dominiczak A, Ebrahim S, Eggermann T, Farrall M, Ferrucci L, Floege J, Forouhi NG, Gansevoort RT, Han X, Hedblad B, Homan van der Heide JJ, Hepkema BG, Hernandez-Fuentes M, Hypponen E, Johnson T, de Jong PE, Kleefstra N, Lagou V, Lapsley M, Li Y, Loos RJ, Luan J, Luttropp K, Maréchal C, Melander O, Munroe PB, Nordfors L, Parsa A, Peltonen L, Penninx BW, Perucha E, Pouta A, Prokopenko I, Roderick PJ, Ruokonen A, Samani NJ, Sanna S, Schalling M, Schlessinger D, Schlieper G, Seelen MA, Shuldiner A.R, Sjgren M, Smit J.H, Snieder H, Soranzo N, Spector T.D, Stenvinkel P, Sternberg M.J, Swaminathan R, Tanaka T, Ubink-Veltmaat L.J, Uda M, Vollenweider P, Wallace C, Waterworth D, Zerres K, Waeber G, Wareham N.J, Maxwell P.H, McCarthy M.I, Jarvelin M.R, Mooser V, Abecasis GR, Lightstone L, Scott J, Navis G, Elliott P, Kooner JS (2010). Genetic loci influencing kidney function and chronic kidney disease. Nat. Genet.

[78] t’Hart LM, Dekker JM, Heine RJ, Maassen JA (2003). Lack of association between gene variants in the ALMS1 gene and Type 2 diabetes mellitus. Diabetologia.

[79] Patel S, Minton JA, Weedon MN, Frayling TM, Ricketts C, Hitman GA, McCarthy MI, Hattersley AT, Walker M, Barrett TG (2006). Common variations in the *ALMS1* gene do not contribute to susceptibility to type 2 diabetes in a large white UK population. Diabetologia.

[80] Arsov T, Silva DG, Obryan MK, Sainsbury A, Lee NJ, Kennedy C, Manji SSM, Nelms K, Liu C, Vinuesa CG, deKretser DM, Goodnow CC, Petrovsky N (2006). Fat Aussie - A new Alström syndrome mouse showing a critical role for ALMS1 in obesity, diabetes and spermatogenesis. Mol. Endocrinol.

[81] Arsov T, Larter CZ, Nolan CJ, Petrovsky N, Goodnow CC, Teoh NC, Yeh MM, Farrell GC (2006). Adaptive failure to high-fat diet characterizes steatohepatitis in Alms1 mutant mice. Biochem. Biophys. Res. Com.

[82] Larter CZ, Yeh MM, Van Rooyen DM, Teoh NC, Brooling J, Hou JY, Williams J, Clyne M, Nolan CJ, Farrell GC (2009). Roles of adipose restriction and metabolic factors in progression of steatosis to steatohepatitis in obese, diabetic mice. J. Gastroenterol. Hepatol.

[83] Teoh NC, Williams J, Hartley J, Yu J, McCuskey RS, Farrell GC (2010). Short-term therapy with peroxisome proliferation-activator receptor-alpha agonist Wy-14,643 protects murine fatty liver against ischemia-reperfusion injury. Hepatol.

[84] Huang-Doran I, Semple RK (2010). Knockdown of the Alström syndrome-associated gene Alms1 in 3T3-L1 preadipocytes impairs adipogenesis but has no effect on cell-autonomous insulin action. Int. J. Obes.

[85] Rüdiger HW, Ahrens P, Dreyer M, Frorath B, Löffel C, Schmidt-Preuss U (1985). Impaired insulin- induced RNA synthesis secondary to a genetically defective insulin receptor. Hum. Genet.

[86] Girard D, Petrovsky N (2010). Alström syndrome: Insights into the pathogenesis of metabolic disorders. Nat. Rev. Endocrinol.

